# Product, not process! Explaining a basic concept in agricultural biotechnologies and food safety

**DOI:** 10.1186/s40504-017-0048-8

**Published:** 2017-03-03

**Authors:** Giovanni Tagliabue

**Affiliations:** Independent researcher, Carugo, Como Italy

**Keywords:** Agricultural biotechnologies, Food safety, GMO

## Abstract

Most life scientists have relentlessly recommended any evaluative approach of agri-food products to be based on examination of the phenotype, i.e. the actual characteristics of the food, feed and fiber varieties: the effects of any new cultivar (or micro-organism, animal) on our health are not dependent on the process(es), the techniques used to obtain it.

The so-called “genetically modified organisms” (“GMOs”), on the other hand, are commonly framed as a group with special properties – most frequently seen as dubious, or even harmful.

Some social scientists still believe that considering the process is a correct background for science-based understanding and regulation. To show that such an approach is utterly wrong, and to invite scientists, teachers and science communicators to explain this mistake to students, policy-makers and the public at large, we imagined a dialogue between a social scientist, who has a positive opinion about a certain weight that a process-based orientation should have in the risk assessment, and a few experts who offer plenty of arguments against that view. The discussion focuses on new food safety.

We are in a food analysis laboratory. The “prototype” of a new variety of sweet pepper has just been put on the table. A few people are there: the lab manager, who is a toxicologist; her assistant, an expert in food-related allergies and intolerance; an agricultural biotechnologist from the philanthropic research unit where the cultivar has been developed; an epistemologist of life sciences who specializes in agri-food biotech regulation and policies. The last attendant is a professor of social sciences, who heads a “Genetic Engineering and Society Center” at a university: since she is convinced that it is necessary to “reboot the debate on genetic engineering”, their discussion is very interesting.

The social scientist starts by affirming that “framing the debate around ‘product versus process’ is neither logical nor scientific” (her statements are quoted from Kuzma 2016, while the other speakers’ lines are imagined) because “from a scientific standpoint, a product’s traits – harmful or otherwise – depend in part on the process by which it is made. (This is especially evident from human gene-therapy trials, where new methods for delivering genes have removed the need for potentially harmful viral vectors.)” The epistemologist strongly disagrees, mentioning a useful categorization of different areas into which the various manipulations of living organisms can be subdivided, using colors as flags: “red” for biomedical-pharmaceutical applications, “green” for agriculture, “white” for industrial, “grey” for environmental bioremediation. The example given by the social scientist refers to the “red” domain, where this or that technique may be significant for a more or less risky outcome; in the “green” biotech world, on the other hand, plants, animals and microorganisms are improved through methods that can be very traditional, like crossing sexually compatible (sometimes even incompatible) varieties and species, or more recently invented, like physical or chemical mutagenesis, or with direct intervention inside the genomes, either by inserting “donor” DNA sequences (transgenesis) or not, through to the present day techniques of gene editing, CRISPR-Cas9 being the latest exciting development (Hall 2016): each method, or a mix of them, cannot be suspected in advance of being more or less problematic and dangerous – or indeed safe. To assess an agri-food product’s risks and benefits, in any imaginable sense, the process(es) which originated it is irrelevant.

The “green” biotechnologist goes on, kindly challenging the social scientist to provide evidence that any technique, or group thereof, is *inherently* unsafe – or safe, for that matter – underlining that no peer-reviewed paper has ever been published that shows how a preemptive caution should be mandatory in any kind of agricultural biotechnology tinkering. However, since any *process* can result in unsatisfactory outcomes, the only rational safety approach is to establish rules for evaluating each individual *product* – the “archetype”, so to speak, of each new variety, to be later multiplied once it has passed muster.

Yet, the lab director recalls that a few genetically engineered cultivars had proved to be very harmful: a squash with toxic properties; a celery made resistant to certain insects, which would bring out rashes in whoever handled it; a very toxic potato, commercial name Lenape (Haslberger 2003, p. 739. Popular article on the Lenape: http://boingboing.net/2013/03/25/the-case-of-the-poison-potato.html). The social scientist quickly seizes her opportunity, underlining that such experiences show how certain processes should be considered significant for stricter regulation! Timidly, the lab director’s assistant says that no, those noxious cultivars were not “GMOs” but unfortunate outcomes from traditional hybridization! With a touch of irony, the biotech expert asks the sociologist whether she thinks that any older method should be strictly regulated, since bad results may emerge… Noting the amused look of the lab director, the sociologist realizes that they have played a little trick on her.

The researcher – this time seriously – points out that also quite a few genetically engineered cultivars came out as unsatisfactory, even unsafe (Haslberger 2003, p. 740; Kuiper et al. 2001, p. 516. See also other examples of “Discontinued Transgenic Products”: http://cls.casa.colostate.edu/transgeniccrops/defunct.html): they were discarded, plain and simple. It happens all the time: breeders, taking advantage of previous experiences and accumulated knowledge, try and see, relying on competence and luck; that’s the way science and technology work, even more so for “green” produce enhancements. In agri-food labs, experimental greenhouses and open field plots, the waste bin is never empty ☹

As the epistemologist emphasizes, it is a basic empirical truth that even the smallest change in the DNA of any organism – obtained via any method – may produce massive phenotypic outcomes, most frequently not beneficial. That’s why life scientists have been preaching for several decades that it is the single *product* of any experiment, not the *process(es)* used to create it, that must be evaluated on its own unique profile of pros and cons related to health, environmental impact and commercial value. Many position statements were issued by several scientific societies all around the world, says the epistemologist; let’s just quote a petition signed by 3,400 scientists – *inter alia* 25 Nobel laureates: “The risks posed by foods are a function of the biological characteristics of those foods and the specific genes that have been used, not of the processes employed in their development.” (Prakash et al. 2000–2014). The same approach has been affirmed with regard to the possible environmental risks of new cultivars: “genetically engineered organisms should be evaluated and regulated according to their biological properties (phenotypes), rather than the genetic techniques used to produce them.” (Tiedje et al. 1989, p. 298). There is probably no other scientific field where the consensus of experts is so overwhelming (Tagliabue 2016): also a few social scientists, who are too often reluctant to admit such general agreement, should come to terms with it.

The actual *process* vs. *product* distinction, the lab director clarifies, relates to the fundamental *ex-ante* vs. *ex-post* approaches. Any risk analysis operations must follow the standards established by the Codex Alimentarius (Codex Alimentarius 2016), which is the international authority set up jointly by the Food and Agricultural Organization (FAO) of the United Nations and the World Health Organization: such detailed criteria dictate the approach to each new food *product* once a breeder submits it (*ex-post*) and does not consider *ex-ante* the technique(s), the *process(es)* used during the experimental manipulations. The new pepper variety that is sitting on the table in front of us may have been derived from mutagenesis, either physical (irradiation) or chemical (exposure to certain substances that change the genome), or from RNA interference, having had some of its traits modified without even changing the DNA structure; it may even come from Mars. The technicians in charge of its safety assessment don’t know and don’t care: their only focus must be on the actual properties of the submitted “prototypes”, following adequate methods for their analyses.

That’s why, the epistemologist points out, the Codex ad hoc committee which was established to write the guidelines for the safety assessment of foods derived from biotechnology (Codex Alimentarius Commission 2003–2008 and 2008) drew up the requested documents, and then was dissolved. Of course, these instructions are a repetition of those to be followed for the evaluation on *any* food, according to different risk profiles, because there is no reason to think that “GMOs” should be treated differently. The committee did its job properly, although it simply repeated that certain kinds of genetic engineering are not *in themselves* riskier than others: a little waste of public money…

The social scientist is not convinced: “In their review procedures, the [regulatory] agencies recognize that the process of engineering is important. The USDA, for example, requires a “detailed description of the molecular biology of the system … used to produce the regulated article”. The biotechnologist’s comment is sharp: while such description may be very interesting for specialists, it has no relevance for the safety of the outcome! Since we know very well that any kind of genotypic manipulation can end up with problematic phenotypic outcomes, why doesn’t the regulator ask for similar procedures in relation to *any* method of genetic manipulation or even for any natural mutation? It would still be pointless, but at least coherent… The lab director nods in approval.

But the sociologist goes on: “Thus, product and process issues are not distinct in regulation. Indeed, it does not make sense scientifically to try to value one approach more highly than the other.” You are both right and wrong, replies the epistemologist: it is true that regulations often result in a (confused) mix of product and process; it is false that both standpoints are similarly correct or useful. Please consider this again: regulating the *process* implies the belief that some kinds of biotech operations must be considered suspicious in advance – and, needless to say, the dubious *products*, as ill-defined as they must be, are the so-called “genetically engineered organisms”. Such bias is unscientific: “The main conclusion to be drawn from the efforts of more than 130 research projects, covering a period of more than 25 years of research, and involving more than 500 independent research groups, is that biotechnology, and in particular GMOs, are not per se more risky than e.g. conventional plant breeding technologies.” (European Commission 2010, p. 16)

“Yet, the sociologist insists, many countries go further than the United States when it comes to process-based triggers for regulation.” “International discussions have focused on which types of gene-editing manipulation fall under regulatory definitions of GE organisms”. The epistemologist now says that we must go back to our high-school memories, recalling the myth of Procrustes: the ancient Greek rogue built a “bed”, i.e. a template where he used to lay his victims, alternatively stretching them or cutting their feet if they did not exactly fit his dogmatic measure. The same arbitrary approach has been embraced by agri-food regulators almost everywhere in the world: they have created a warped frame inside which they struggle to pigeon-hole the results of certain *processes* (“GMOs!”) even before asking the question – the only rational one – whether this or that *product*, individually considered, is more or less safe or unsafe. Their ongoing embarrassment when new techniques emerge that cannot be placed inside, or outside, their nonsensical rickety fence, is not surprising (Tagliabue 2015).

Plain and simple, says the biotech expert, law-makers have been replicating an ongoing big mistake, because what we need is not groundless pre-emptive wariness in regard to this or that “green” biotechnique (*ex-ante*), but accurate examination of each “prototype” – such as our pepper on the table – once created and selected from many unlucky outcomes (*ex-post*): if it is safe, hooray!, we will clone and propagate it in millions of copies to be sold on the market; in many parts of the world, it will be crossed with local varieties, well adapted to different climates and soils. Alas, not every new cultivar will be available soon and everywhere, the lab director reminds us: if it is a “GMO”, in many countries there will be lots of added controls, never-ending red tape, legal hurdles, inflated costs or even straight prohibition. (McHughen 2016)

But the sociologist declares that even life scientists think that certain processes have to be discussed regarding their possible sectoral regulation: “Ironically, the same GE developers who once claimed that the process of GE does not matter for regulatory purposes are now arguing that changes to the engineering process justify looser regulatory scrutiny.” That’s because, argues the epistemologist, these breeders are desperately trying to evade the “GMO” semantic trap and the related regulatory thicket: a thorough overhaul of the present legal background, in which a motley bunch of processes and resulting products wear a scarlet letter, is very unlikely for the time being; therefore, “green” biotechnologists are hoping that the new techniques will escape the nonsensical added rules that have been overburdening an ill-defined pseudo-category of things!

The social scientist adds that “product-based arguments lead to one of two conclusions: if all products (GE or otherwise) are to be treated the same, then either all products – GE and conventionally bred – should be regulated, or neither should be. The first option is impractical and the second inadvisable given that some products could be harmful.” What do you mean “impractical”, asks the lab director: since we know that any new *product*, independently of the *process* applied to create it, may be harmful, each new agricultural invention should be evaluated on its own. Even if we manage to get rid of any pointless preemptive regulation, the golden rule to be applied would remain the same, that is to check out any new organism: this is not “inadvisable”, just the opposite! Instead, you know what?, “GMOs” are over-scrutinized, while all the others are often not properly controlled. For instance, the EFSA (European Food Safety Authority), i.e. the EU agency in charge of scientific risk assessment, has a section which is explicitly dedicated to “GMOs”, with a goodly number of scientists on the expert panel, detailed guidelines and precise instructions for breeders who want to submit an application for the authorization of a rDNA-derived product (www.efsa.europa.eu/en/science/gmo); very similar new foods which are obtained with slightly different biotech methods are completely exempt, and are not even mentioned. Where’s the rationale for this?

If I may, the assistant chimes in, the approach aiming to regulate the “green” biotech *process(es)*, instead of the actual characteristics of each *product*, results in really bizarre consequences: I can offer two examples, among many. 1. One of the most common traits obtained via recombinant DNA is crop tolerance to herbicides: farmers find it convenient to buy seeds of maize or soybean or other crops coupled to a weed-killer substance that prevents weed growth but leaves the valuable plants intact. However, there is another series of varieties of the same crops (some are for instance traded under the commercial name Clearfield) that, similarly, have been made tolerant to proprietary herbicides, and consequently are used together with them; but the desired trait has been obtained through tissue culture and/or induced mutagenesis or selection of natural mutants which are then crossed with other varieties and so – legalistically speaking – without creating “GMOs”: consequently, the producer has legitimately and happily avoided the very major barriers which it would have had to face if the technical staff had directly pinched the DNA of the new varieties of wheat, rice, sunflower, lentil, corn and canola (BASF 2017). Thus, similar cultivars made tolerant to weed killers through direct genetic transfer (“GMOs”!) are subject to endless risk analyses, while for other products, in which it has been possible to express an identical trait, by modifying the genome in different yet targeted ways, the burdensome tests are not imposed. 2. Even more curious is the case of the Amflora potato (VIB 2010, http://en.wikipedia.org/wiki/Amflora). It was genetically engineered in order to inhibit the production of one of the two kinds of starch which are typically present in the tuber. This modification is useful for many industrial applications (e.g. the production of paper, which absorbs a large share of potatoes) and avoids a costly and polluting process: the inactivation of a certain gene solves the problem at source. The push and shove between the European Commission, the ministers of various recalcitrant European states, and the challenges of “anti-GMO” organisations concerning authorization of the new cultivar lasted 17 years and eventually convinced the producer to give up marketing the product in Europe (Laursen 2012), only to see insult added to injury: another German company managed to produce the same desired phenotypic trait through a “non-GMO” method of mutagenesis, and started the mass production of its “Super potato” without any particular bureaucratic burden (Fraunhofer-Gesellschaft 2009). No logic whatsoever in regulating the *processes* instead of the *product*!

Generally speaking, the lab director continues, we can see again a confusion between two very different concepts and related procedures: “*ex-ante* regulation” vs. “*ex-post* controls”. In a rational world, preemptive regulation is intended to set the legal frame with regard to harmful *processes*. Remember the “red” biotechnologies? When we are dealing with infective viruses or lethal bacteria, we must be a priori very, very careful: strict safety rules are a must, since any mistake in handling pathogens can have dire consequences. In the “green” area, we perform the controls *a posteriori*, carefully examining single *products* like this promising new pepper variety. Of course, that’s the reason why in my lab nobody is wearing safety garments: we are testing eggplants and harmless enzyme-producing bacteria, not vaccines.

This is why, the epistemologist adds, some images which are frequently shown as a complement to articles regarding agri-food “GMOs” in the media are meaningless and misleading: photos of people in maize fields with suits reminiscent of bacteriological warfare are ludicrous – yet impressive (Clancy and Clancy 2016), quite effective in terms of “anti-GMO” scaremongering.

Therefore, the new foods developer says to the sociologist, you have already drawn the correct conclusion – which may be surprising for laypersons and quite a few social scientists, not for geneticists, biologists and breeders: as far as agricultural products are concerned, no special precautionary safety regulation is necessary for the *processes*, while controls should be performed on each novelty (*product*), case by case, obviously before starting mass production. Note again that the present situation is very unbalanced almost everywhere: obsessive tests and redundant analyses are mandatory for “GMOs”, almost no supervision is requested for the “prototypes” of all the other products.

I anticipate a possible objection, says the lab director: isn't it risky to rely on an *ex-post* criterion of controls, given that some inadequate, or even harmful, trait can escape scrutiny? Well, some risks may always exist. But, again, we must be aware that a higher or lower level of possible unsafety is *not* related to the process(es) used to create new cultivars: therefore, any *ex-ante* regulation of agri-food biotechnique(s) does not add anything interesting or useful to the safety issue.

Thus, says the biotechnologist, reading quotes from an article written by the sociologist, asking “what classes of GE products or processes should receive greater regulatory scrutiny” is simply a groundless question if related to the agri-food area, and the alleged “need to consider a mix of product and process issues to capture product groups that are likely to be of greater concern” is utterly misplaced.

The sociologist poses another problem: “it is impossible to be completely ‘science based’ in a regulatory system. Value judgments are embedded in all risk and safety assessments. For example, the dose–response curve for a certain food additive might be known, but such data do not by themselves tell regulators where to set an acceptable safety limit.” There is a chilly moment: the lab director and the epistemologist stare at each other. Please, colleague, the latter slowly says, be aware that you are confusing *risk assessment* and *risk management*: the first is definitely a technical issue, the second is a policy matter – although decisions (management) should be based on the best scientific evidence available (assessment). This is a bedrock of the guidelines for risk analyses, as accepted at the widest international level: “There should be a functional separation of risk assessment and risk management, in order to ensure the scientific integrity of the risk assessment, to avoid confusion over the functions to be performed by risk assessors and risk managers and to reduce any conflict of interest.” (Codex Alimentarius 2016, p. 125). We are not talking about watertight compartments, since the same basic text establishes the prominence of risk managers in giving preliminary instructions on how to conduct the risk assessment: “Risk assessment policy should be established by risk managers in advance of risk assessment, in consultation with risk assessors and all other interested parties.” (Codex Alimentarius 2016, p. 126). We all know that our knowledge is still relative, sometimes poor: for example, the toxicity of asbestos was undervalued for a long time! But the (scientific) *assessment* of the risks, even if necessarily shaky or incomplete, is the starting point for deciding the (political) *management* of it: how could it be otherwise?

In a seminal text (NAS-NRC 1983), the epistemologist points out, a clear indication was given on keeping assessment and management separate, and the reason was the frequent political interference in the scientific activity of the Environmental Protection Agency in the previous years: this situation was criticized by many. Maybe the sociologists who have been insisting for several decades regarding the alleged blurry border between risk assessment and management are obtaining the undesired effect of encouraging the politicization of risk assessment? That would be ironic. I think it’s time to reconsider the insistence of many social scientists on the near-impossibility of neutrally scientific risk assessment guidelines, procedures and tests as a sort of intellectual infatuation. In this sense, a more sober and sobering approach may be imagined, e.g. in terms of a limited return to the “bureaucratic expertise model”. (Marchant 2012)

In labs like mine, adds the director, we are in charge of evaluation of new agricultural products, as far as possible toxicity (www.fda.gov/food/guidanceregulation/guidancedocumentsregulatoryinformation/ucm081825.htm) or allergenicity (USA: http://www.jaad.org/article/S0190-9622(10)02126-2/abstract; UK: www.nice.org.uk/guidance/CG116) effects are concerned: and let me tell you, although we are not omniscient, our tools and procedures *are* “science-based”, while we follow the risk assessment policy as established by risk managers.

In the meantime, the lab director’s assistant has put a couple of items on the table and he now briefly explains what they are. 1. Seeds of *Scuba rice*, created at the International Rice Research Institute in the Philippines, with the help of the University of California at Davis: this innovative variety is good in the event of flooding, a frequent occurrence with Asiatic monsoons; the seeds have been distributed to several millions of small farmers, mostly in India. The gene which allows the new cereal to withstand prolonged immersion was found in a landrace and transferred into high yielding modern varieties; interestingly, the preliminary experiments designed to confirm that gene was the correct one required the creation of a “GMO”: the DNA sequence was introduced by transgenesis into the genome of a model variety of rice. Once the effectiveness of the gene was confirmed, it was then moved by conventional crossing (Xu et al. 2006) accelerated by Marker Assisted Selection. The non-transgenic method was chosen, despite being slow and less precise, only to avoid wasting years with the bureaucratic nightmare which oppresses “GMOs”. (Prof. Pamela Ronald, personal communication) 2. This small box, the lab assistant continues, contains a variety of yeast made more efficient through the technique nicknamed *Delitto perfetto* (Storici and Resnick 2006), in which donor DNA sequences are inserted and then cancelled, once a satisfactory phenotype has been obtained for subsequent multiplication: a *product* that leaves no traces of the *process*! The safety – or otherwise – of the rice and the yeast, both organisms enhanced through human ingenuity, that our lab has been requested to assess, has nothing, *nothing* to do with the mix of methods concocted by the developers’ imagination.

Now the lab director shows a bottle of maize syrup: our technicians have fully examined it, she says, the *product* is safe and nutritious. It contains no proteins, no DNA; even with our advanced test tools, we cannot say whether it comes from transgenic cobs or otherwise – the *process*. If somebody asks me whether it is a “GMO” or not, I will kindly reply that such a question is meaningless – although the temptation would be to break the bottle on their head…

The epistemologist looks the now impressed sociologist directly in the eye, saying: if these examples do not delete in your mind the very notion of the supposed relevancy of the *process(es)* to assess the qualities – or defects – of a *product*, we’ll be very disappointed… ☹

Therefore, the biotech expert hopes to conclude, we don’t need to “reboot the debate on genetic engineering”, starting once again on the wrong foot. All we need is to follow a rational, *science-based* path, applying the necessary scrutiny to each and every new agri-food product – “GMO” or otherwise – before its multiplication and commercialization.

The epistemologist adds that if lawmakers wanted to reconsider the existing “green” biotechnology regulations, which are almost always warped, a rational and *science-based* technical-legal framework is already available (Conko et al. 2016); it is the result of the participatory work of a number of scientists from several countries. The guidelines for careful assessment of new cultivars according to classes of gradual risk are explained; to ascertain the pros and cons of each new plant, the different biotech methods are considered irrelevant: the “GMO” blunder is not even mentioned! As for field tests, sensible questions are asked, regarding the ecological impact (to what extent is the plant potentially invasive?) and the health issues (what tests need to be performed to evaluate possible allergenicity or toxicity?). Please note that we are talking about assessing and listing the safety controls to be performed *ex-post*, i.e. when each agri-food invention has been put on the lab table. The creators of these guidelines emphasize that it is not a mere theoretical hypothesis, but it draws inspiration from similar experiences which are already well tested in the real world: it is analogous to existing regulatory regimes, such as those for quarantine regulations for plant or animal pests. The approach is not fundamentally new and has worked well in practice for decades.

Therefore, the biotechnologist declares with a slight but evident provocative tone, the public should not be lured into a frequent delusion: the pressing problem is not the relationship between “GMOs” and society, because this is a misleading approach.

The social scientist is now a little upset: should I shut down my “Genetic Engineering and Society Center” then, she asks?! No ma’am, the epistemologist quietly replies, in our humble opinion you should reassess the scope of your department, making it a “Biotechnologies and Society Center”: this way, if you allow us, the narrow focus on the botched “GMO” pseudo-category would be widened to a real societal big issue…

P.S. – One year after, bad news: the delicious new pepper variety, as good and safe as it was, has given unsatisfactory yield results, some 15–20% lower than its competitors which are already on the market. At the research department, after a moment of depression, breeders are ready to try again: one group of developers thinks that a hybridization with an “heirloom” cultivar of pepper may be successful, adding better organoleptic qualities to high productivity; another group imagines that inserting into the pepper’s genome a certain DNA sequence taken from an orchid could do the trick. Hopefully, in a few months they will show up again at the food analysis lab to assess the safety of some new prototypes. There, technicians will perform the relevant tests again, without even asking how the new *product* has been obtained: everybody knows that the *processes* are irrelevant.

P.P.S. – This is just a fantasy, because the first outcome of the next experiment – the hybridized cultivar – would be quickly examined, while the second approach would create a “GMO” and therefore start a long, costly path.
